# Hypothesis-driven methods to augment human cognition by optimizing cortical oscillations

**DOI:** 10.3389/fnsys.2014.00119

**Published:** 2014-06-26

**Authors:** Jörn M. Horschig, Johanna M. Zumer, Ali Bahramisharif

**Affiliations:** ^1^Radboud University Nijmegen, Donders Institute for Brain, Behaviour and CognitionNijmegen, Netherlands; ^2^School of Psychology, University of BirminghamBirmingham, UK

**Keywords:** neuronal oscillations, working memory, attention, elderly, ADHD, brain stimulation, brain-computer interfacing, brain state dependent tasks

## Abstract

Cortical oscillations have been shown to represent fundamental functions of a working brain, e.g., communication, stimulus binding, error monitoring, and inhibition, and are directly linked to behavior. Recent studies intervening with these oscillations have demonstrated effective modulation of both the oscillations and behavior. In this review, we collect evidence in favor of how hypothesis-driven methods can be used to augment cognition by optimizing cortical oscillations. We elaborate their potential usefulness for three target groups: healthy elderly, patients with attention deficit/hyperactivity disorder, and healthy young adults. We discuss the relevance of neuronal oscillations in each group and show how each of them can benefit from the manipulation of functionally-related oscillations. Further, we describe methods for manipulation of neuronal oscillations including direct brain stimulation as well as indirect task alterations. We also discuss practical considerations about the proposed techniques. In conclusion, we propose that insights from neuroscience should guide techniques to augment human cognition, which in turn can provide a better understanding of how the human brain works.

## Introduction

Recent advances in cognitive neuroscience have provided insight into the functional mechanisms of the human brain. Neuroscientists have identified specific brain patterns, for example neuronal oscillations, that co-fluctuate with the task and behavioral performance (Buzsáki, 2006). These fluctuations are not random but depend on the specific task and cognitive settings; these findings have allowed functional hypotheses to be formed, directly tested, and confirmed. Throughout the previous decades, huge progress has been made in understanding how the human brain works, and in understanding differences across age groups, pathologies, and individuals. Applying this in-depth knowledge in practice might therefore be a key to creating brain tools for different target groups to improve different aspects of human cognition.

Cognitive functioning declines with age (Deary et al., 2009) and does not necessarily occur in the presence of a neurological disorder. Healthy elderly suffer from problems with memory and attention more than healthy, young individuals. With an increasing aging population, it has become a societal priority to look into approaches that can delay or prevent functional degeneracy or even augment cognitive abilities in the elderly. Brain tools might have the potential to rejuvenate the functionality of an aging brain.

Many people suffer from cognitive deficiencies in daily activities, but there are population groups in which these problems are more severe. Attention deficit/hyperactivity disorder (ADHD) is a well-studied disorder with problems of attention, hyperactivity, and impulsivity. Although the cause of ADHD is unknown, there have been many attempts to treat it using medication (e.g., Chang et al., 2012). Next to many unknown side-effects of medication, about 30% of the ADHD population do not respond to any medication, which calls for alternative treatments (Kidd, 2000). Brain tools might serve this population by normalizing their brain activities.

Most of our knowledge from cognitive neuroscience about the human brain stems from studies on healthy, young individuals, which have helped to form functional hypotheses about traits of human brain activity. These hypotheses can serve as a benchmark for other populations groups. In addition, also healthy young adults show large task variability in cognitive tasks. Next to individual differences, individuals’ performance varies momentarily in cognitive tasks (Kane and Engle, 2002; Paulus et al., 2009). Thus, while constituting a proper control group, we will also discuss our current knowledge on whether young, healthy adults can benefit from cognitive improvements.

The brain is a highly flexible organ which can adapt to different manipulations very quickly (Pascual-Leone et al., 2011). Entrainment of neuronal oscillations to augment human behavior has already been proposed in the past (see e.g., Thut et al., 2011a; Herrmann et al., 2013; Calderone et al., 2014; Enriquez-Geppert et al., 2014). We complement these reviews by proposing different techniques in different target groups to relate the to-be-augmented aspect of cognition to associated neuronal signatures, specifically neuronal oscillations. Identifying the neural signatures of different tasks will allow for proposing protocols for manipulating the brain and thereby the individual’s cognitive abilities. Recent studies suggest a causal role of neuronal oscillations in cognitive tasks (Thut and Miniussi, 2009; Romei et al., 2010). Based on this hypothesis and the possibility of manipulating neuronal oscillations in several ways, we propose that by using “hypothesis-driven” approaches, one can augment human cognition by optimizing cortical oscillations. In this paper, we begin with discussing the functional role of neuronal oscillations and their cognitive relevance. We then continue with more details about three target population groups, healthy elderly, patients with ADHD, and healthy young adults, and elaborate on how cognitive improvement can be gained. Next, we go into different ways of manipulating functional oscillations in order to improve cognitive performance in the three target groups. The paper ends with practical considerations and conclusions.

## Functional role of neuronal oscillations

Spontaneous and goal-related fluctuations of the brain state are reflected in electrophysiological activity that can be measured non-invasively using various techniques like electroencephalography (EEG) and magnetoencephalography (MEG). EEG and MEG measure the strength of the voltage potentials and magnetic fields at the scalp associated with postsynaptic potentials along the dendrites of pyramidal neurons, i.e., the synaptic input to these cells (Nunez, 2000; Niedermeyer and Lopes da Silva, 2005; Wang, 2010; Lopes da Silva, 2013). Non-invasive measurements require strongly synchronized activity across nearby neurons to result in a measurable signal at scalp level. Neuronal oscillations at the scalp level are rhythmic patterns that represent the degree of synchronized neuronal input to the underlying neuronal ensemble (Lopes da Silva, 1991; Buzsáki and Draguhn, 2004), which are reflected as power increases (commonly known as event-related synchronization, ERS) or power decreases (event-related desynchronization, ERD; see Pfurtscheller and Lopes da Silva, 1999).

Neuronal oscillations are commonly divided into different frequency bands. While lower frequencies are often associated with long-range connectivity between cortical regions (von Stein and Sarnthein, 2000), higher frequencies reflect the local firing pattern of neurons (Xing et al., 2012). Furthermore, different neuronal oscillations have been associated with specific neuronal processes (e.g., Engel et al., 2001; Kopell et al., 2010), which have been related to behavioral performance, e.g., in attention and working memory tasks (reviewed in more detail below). In this paper, we will focus on cortical oscillations in three frequency bands: alpha oscillations (8–13 Hz), theta oscillations (5–8 Hz), and gamma oscillations (>30 Hz). In the following, we will introduce these three oscillations, describe the current dominant views on their function, and elucidate their role on qualitative aspects of cognition.^1^

### Alpha oscillations (8–13 Hz): functional inhibition of neuronal regions

Human alpha oscillations are the dominant rhythms in EEG and MEG. They were observed almost a century ago (Berger, 1929; Adrian and Matthews, 1934) and, while originally having been associated with the idle state of the visual cortex, the current dominant view has changed towards a functional, inhibitory role, elaborated upon below. Alpha is generated via thalamocortical and cortico-cortical loops (Lopes Da Silva and Storm Van Leeuwen, 1977; Lopes da Silva et al., 1980; Suffczynski et al., 2001; Bollimunta et al., 2011). Cortical alpha has been shown to be modulated by the pulvinar nucleus of the thalamus (Saalmann et al., 2012) as well as by frontal regions (Capotosto et al., 2009). The precise mediation of cortical alpha via the interplay between frontocortical and subcortical mechanisms still requires further investigation. Individual variations of alpha power appear as stable traits. The peak frequency of alpha oscillations has been related to the latent factors of general cognitive abilities, and the frequency and power of alpha oscillations have been shown to change with age (Klimesch, 1999; Grandy et al., 2013).

Alpha oscillations are strongly involved in attention processes. While low alpha activity can be observed in regions that are processing information, sensory regions that are not involved in the current task show high alpha activity. For example, in covert spatial attention studies, selective attention and successful inhibition of the task-irrelevant hemifield are often indexed using the extent of alpha power lateralization. Highly lateralized alpha activity thereby suggests a high inhibition of the task-irrelevant hemifield and has been shown to lead to better task performance (Worden et al., 2000; Thut et al., 2006; Kelly et al., 2009), whereas a weak or reverse alpha lateralization leads to failures in motor inhibition (Bengson et al., 2012). During the retention interval of working memory tasks, it has been shown that the degree of alpha power increase in early sensory regions scales with memory load (Jensen et al., 2002). This effect also holds true for paradigms in non-visual domains, e.g., occipital alpha increases during a somatosensory delayed-match-to-sample task (Haegens et al., 2010; Spitzer and Blankenburg, 2012). Additionally, strong alpha during the immediate rehearsal of an item for long-term memory encoding has been shown to predict the long-term memory encoding success (Meeuwissen et al., 2011). Alpha activity has therefore been suggested to reflect the amount of top-down controlled cortical inhibition (reviewed in Klimesch, 1999, 2012; Klimesch et al., 2007; Jensen and Mazaheri, 2010; Foxe and Snyder, 2011; Jensen et al., 2012).

**Figure 1:**
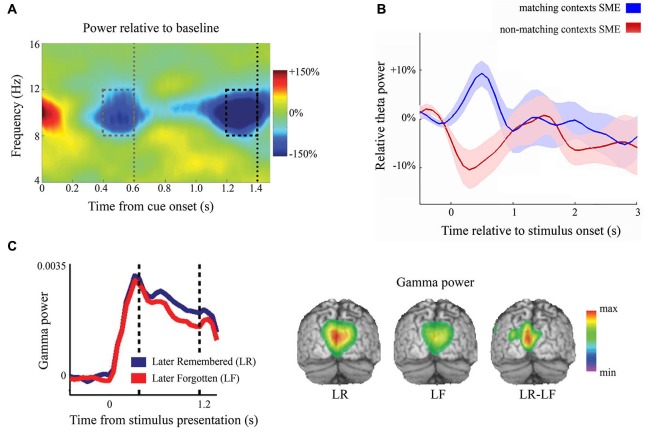
**Example of the functional involvement of neuronal oscillations. (A)** The time-frequency representation of an anticipation task. The gray dotted line shows the time that the stimulus could have been presented, but was not. The black dotted line indicates the onset of the target. Alpha power decreases in anticipation of a stimulus even without subsequent stimulation. Reproduced with permission from Rohenkohl and Nobre (2011). **(B)** The dynamics of frontal theta power. Depending on the encoding task, both increases and decreases of frontal theta power were found for successful versus unsuccessful remembering. SME = subsequent memory effect. Reproduced with permission from Hanslmayr and Staudigl (2014). **(C)** During visual stimulus encoding, posterior gamma power (60–90 Hz) is predictive of subsequent memory performance in young, healthy adults. The vertical dashed bars indicate the window for significance testing and beamformer application. The left panel shows gamma power from one significant posterior sensor and the right panel is the beamformer projection (Osipova et al., 2006).

Alpha oscillations are also strongly involved in the anticipation of upcoming stimuli, which has been convincingly shown by Rohenkohl and Nobre (2011). They showed that when stimulation was temporally anticipated but absent, alpha power decreased to a similar degree compared to when stimulation actually occurred when anticipated, see Figure 1A. Moreover, the degree of anticipatory alpha power correlates with subsequent behavioral performance in attention tasks (Worden et al., 2000; Thut et al., 2006; Kelly et al., 2009) and correlates with subsequent long-term memory performance (Park et al., 2014). Anticipatory alpha power also scales with stimulation likelihood (Gould et al., 2011; Haegens et al., 2012; Horschig et al., 2014). Upon stimulation, however, alpha oscillations robustly decrease in early sensory regions. Recently, Hanslmayr et al. (2012) suggested an entropy-based explanation for this: the more complex the information is that needs to be encoded, the less structured (i.e., the more de-synchronized) the activity in a network has to be. An alternative explanation, which is not mutually exclusive, is that early sensory regions become dis-inhibited, i.e., functionally engaged, and therefore show low alpha power.

### Theta oscillations (5–8 Hz): working memory and neuronal organization

Depending on where in the brain theta oscillations are observed, they can be divided into two groups of hippocampal and cortical theta rhythms (reviewed in Mitchell et al., 2008). In this paper we only focus on the cortical theta rhythms that can be measured non-invasively and are probably generated in hippocampal-cortical feedback loops (Klimesch, 1999). Cortical theta power has been related to encoding, retaining, and retrieving items in working memory (Kahana et al., 2001; Klimesch et al., 2010; Sauseng et al., 2010). The frequency of theta rhythms shows a large inter-individual variability similar to alpha oscillations, and the individual theta peak frequency has been shown to be significantly correlated to the individual alpha peak frequency (Klimesch et al., 1996).

During working memory tasks, theta power increases over temporal sites during encoding, maintenance, and retrieval (Raghavachari et al., 2001; Fell et al., 2011). Over frontal regions, theta power increases proportionally with task demands (Gevins et al., 1997). In working memory tasks, for example, a higher memory load produces stronger frontal theta activity (Jensen and Tesche, 2002). However, both frontal theta increases and decreases have been found to be beneficial for successful memory retrieval (Staudigl and Hanslmayr, 2013; see Figure 1B). Hanslmayr and Staudigl (2014) recently suggested that the context of the memory and the probe items is the crucial factor for whether theta increases or decreases upon successful retrieval. Raghavachari et al. (2006), however, argued for local theta generators where frontal theta exerts executive control and parietal theta serves to maintain items in working memory.

Apart from their role in working memory, frontal theta oscillations link prediction errors to behavioral adaptations (Cavanagh et al., 2010). Theta oscillations are also involved in long-range neuronal communication between cortical and subcortical regions including the hippocampus (Mitchell et al., 2008) and nucleus accumbens (Cohen et al., 2009, 2012) as well as for corticocortical communication (reviewed in von Stein and Sarnthein, 2000). In conclusion, contemporary theory posits that theta oscillations are crucially involved in the organization and coordination of information and for memory storage and retrieval (Jensen and Lisman, 1996; Buzsáki and Draguhn, 2004; Lisman, 2005, 2010; Sauseng et al., 2010; Lisman and Jensen, 2013).

### Gamma (>30 Hz): perceptual and multisensory binding and memory maintenance

Gamma oscillations are defined as frequencies above 30 Hz, subdivided into the lower gamma range (30–80 Hz) and the higher gamma range (>80 Hz; Buzsáki and Draguhn, 2004; Ray and Maunsell, 2011). Gamma oscillations have been implicated with active processing of information and thus increase with stimulation intensity and amount of attention to stimulation (Engel and Singer, 2001; Engel et al., 2001; Fries et al., 2001; Jensen et al., 2007). A large inter-individual variability in the gamma peak frequency has been demonstrated (van Pelt et al., 2012) as well as a correspondence between the gamma peak frequency and the characteristics of the visual stimuli (van Pelt and Fries, 2013).

Gamma oscillations are also observed during working memory maintenance (Tallon-Baudry et al., 1998; Miltner et al., 1999; Jokisch and Jensen, 2007), possibly reflecting active processing and binding of the to-be-maintained information in frontal and parietal cortices (Polanía et al., 2012b). The degree of gamma power during encoding of visual items has been found to correlate with working memory load (Howard et al., 2003) and predicts successful memory encoding, see Figure 1C (Osipova et al., 2006). Also, during successful multisensory integration, an increase of gamma band power has been observed (Schneider et al., 2008; Kanayama et al., 2012), strengthening the idea that gamma band oscillations serve to form a coherent object representation in working memory. Gamma band activity has been suggested to reflect the process of mentally forming and binding objects (Tallon-Baudry et al., 1996; Tallon-Baudry, 1999).

Many studies report a simultaneous decrease of alpha power and increase in gamma power in task relevant brain regions, whereas a number of studies have shown that alpha and gamma power are not always inversely coupled (Haegens et al., 2010; Scheeringa et al., 2011). For example, while alpha power has been shown to decrease in anticipation of a stimulus (Rohenkohl and Nobre, 2011; see Figure 1A), gamma activity is induced by stimulation and active maintenance but is not observable in anticipation to stimulation (Hoogenboom et al., 2006). Thus, while alpha decreases may serve to disinhibit a brain region, gamma oscillations reflect the active, ongoing processing and binding of information.

## Population target groups and their neuronal signatures

Many common individual differences in cognition can be traced back to differences in memory or attention processes. In the previous section we outlined the functional role of neuronal oscillations in cognition and presented strong evidence for a relationship between oscillatory power and behavioral performance in specific tasks. In this section, we will outline how optimizing neuronal oscillations might improve cognition in three target groups: the elderly, who commonly have problems with attention and working memory, patients suffering from ADHD, and also healthy, young adults who show remarkable inter-individual differences in cognitive tasks. We will propose that optimizing neuronal oscillations might help to alleviate symptoms and improve cognition.

### The elderly and their problems with attention and working memory

In the western world, there is a continuous demographic change with an increased percentage of elderly people in the population (Cohen, 2005; Peters et al., 2010). Several studies have statistically assessed the areas of compromised cognition in the elderly. Elderly people have more trouble in task switching paradigms compared to younger adults (Kray and Lindenberger, 2000). The elderly show lower performance in working memory tasks (Salthouse et al., 1991) and have reduced working memory capacity (at least in part) due to problems in binding multiple low-level features (Brockmole and Logie, 2013). Additionally, it has been shown that elderly have trouble inhibiting distracting information in unimodal tasks (Folk and Lincourt, 1996; Groth and Allen, 2000; Gaeta et al., 2001; Tales et al., 2002; Andrés et al., 2006; Fabiani et al., 2006; Rowe et al., 2006; Yang and Hasher, 2007), in cross-modal tasks (Alain and Woods, 1999; Poliakoff et al., 2006; Hugenschmidt et al., 2009b), and multi-modal tasks (Hugenschmidt et al., 2009a). The studies by Hugenschmidt et al. and others suggest that while elderly have trouble ignoring task-irrelevant items, they do show intact attention abilities. For example, elderly showed behavioral cueing effects, even in more complex environments (Hugenschmidt et al., 2009a,b).

In addition, a number of structural and functional changes have been reported in the brain of the elderly. For example, with increasing age, alpha peak frequency and power decrease while theta power increases (Dustman et al., 1993; Grandy et al., 2013) and the amount of evoked gamma band activity is reduced in the elderly compared to young adults (Werkle-Bergner et al., 2009). Recently, Sander et al. (2012a) proposed to disassemble working memory into two components: a global top-down control mechanism and a local perceptual binding mechanism. They further hypothesized that both components are differentially impaired in the elderly. By incorporating the reviewed evidence from the previous section, global top-down control is reflected by the power of frontal theta and posterior alpha oscillations (reviewed in Klimesch, 1999; Klimesch et al., 2007; Jensen and Mazaheri, 2010; Foxe and Snyder, 2011; Jensen et al., 2012; Klimesch, 2012), whereas local binding is reflected by gamma band oscillations (Tallon-Baudry et al., 1996; Tallon-Baudry, 1999). Under this framework, reduced alpha or theta power would suggest a problem with top down control, whereas a reduction in evoked gamma-power would predict a problem with perceptual binding.

**Figure 2:**
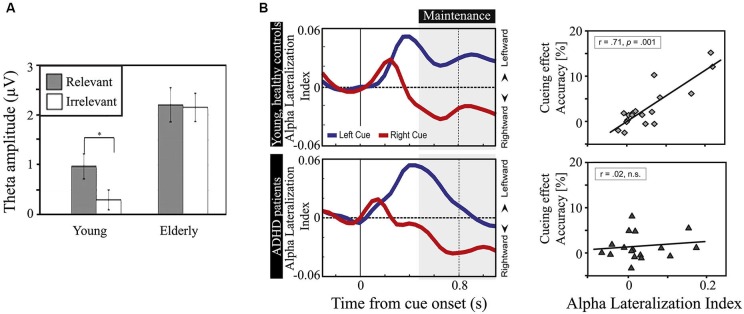
**Oscillatory power differences in different task settings in the three discussed population groups. (A)** Frontal theta power during stimulus encoding is significantly higher for elderly versus young adults, and scales with relevance of the stimuli in young, healthy adults, but not in the elderly (* *p* < 0.001). Reproduced with permission from Gazzaley et al. (2008). **(B)** During visual spatial covert attention, the degree of alpha lateralization indexes the relative disengagement of the task-irrelevant ipsilateral hemisphere versus the task-relevant contralateral hemisphere. While young, healthy adults are able to modulate their alpha lateralization symmetrically around zero, inattentive ADHD patients show a lack of maintaining a high degree of alpha lateralization to the left side. In addition, there is an absence of correlation between alpha lateralization index and behavioral cueing effect in ADHD patients, whereas there is a strong relationship in healthy, young adults. Reproduced with permission from ter Huurne et al. (2013).

A number of studies showed the absence of correlations between behavior and oscillatory activity in the elderly, while present in healthy young adults. For example, a strong increase in frontal theta power with working memory load has been reported in young adults (Jensen and Tesche, 2002), but there was no such relation in the elderly (McEvoy et al., 2001). Also, McEvoy et al. (2001) found that with increasing task difficulty, alpha power in both parietal and frontal cortices decreased in the elderly, whereas in young adults alpha power only decreased over parietal regions. In a similar vein, Gazzaley et al. (2008) showed that frontal theta power scaled with the relevance of the to-be-processed item in young healthy adults, but not in the elderly, see Figure 2A. Sander et al. (2012b) showed that in a visual covert attention working memory paradigm, the degree of lateralized alpha power was maximal under high memory load in healthy, young adults, whereas it peaked under medium memory load in the elderly and was nearly absent in the high memory load condition. Despite the absence of this correlation, the elderly were still able to successfully perform well in this task, although worse than the young adults.

All studies in elderly are fraught with the problem that compensatory mechanisms seem to be active (Reuter-Lorenz et al., 2000; Cabeza et al., 2002; Logan et al., 2002; Nielson et al., 2002; Riis et al., 2008). While a reduced amount of activity might highlight the locus of the problem, an increase in activity elsewhere might indicate the compensation. Hence, in the above finding by Sander et al. (2012b) it is likely that a compensatory mechanism took over the functional role of posterior alpha lateralization under high memory load. Identifying such mechanisms and studying whether they are compensatory or competing with normal functioning could help in determining whether this mechanism is causing or resulting in the degraded bottom up sensory processing and binding.

### Attention deficit/hyperactivity disorder

ADHD is the most common psychiatric disorder in the western world (Cantwell, 1996; Barry et al., 2003) with an estimated prevalence of 3–6% (Pelham et al., 1992; Polanczyk et al., 2007). Diagnosis of ADHD is characterized by two components: an attention and a hyperactivity component. While some patients show traits of both components (the “combined” subtype), a large proportion of patients show only one component strongly with the other component weaker or absent (Barkley et al., 1990; Lockwood et al., 2001). Here, we will focus on the combined and the inattentive subgroups, i.e., those with attention deficits. However, the combined patient subgroup is characterized by different attention deficits in cognitive tasks than the inattentive subgroup (Weiss et al., 2003; Booth et al., 2007; Adams et al., 2008), leading to the proposition of categorizing patients showing inattentive without hyperactivity symptoms as a patient group distinct from ADHD (Barkley, 2001; Milich et al., 2001; Derefinko et al., 2008). Nonetheless, we will discuss characteristics and possible treatments for attention deficits of both the combined and the inattentive subgroups.

The inattentive subtype is characterized, as the name implies, by problems in engaging and sustaining attention. In behavioral paradigms it has been found that the inattentive subgroup showed either reduced attention resources (Carr et al., 2010) or reduced visual processing power (Weiler et al., 2002). The inattentive subtype also showed a lack of response cueing effect (Lockwood et al., 2001; Derefinko et al., 2008). Behavioral deficits and oscillatory power differences between ADHD patients and control subjects have been investigated recently. In adolescents, Mazaheri et al. (2013) found that ADHD patients of the inattentive subgroup showed a reduced behavioral cueing effect and weaker suppression of posterior alpha in response to the cue that indicates with which hand to respond. In young adults with the inattentive subtype, ter Huurne et al. (2013) showed an absence of maintaining posterior anticipatory alpha lateralization in response to a left attention cue, but not to a right attention cue. Interestingly, they found the same initial level of alpha lateralization in control and ADHD groups. In both studies, the degree of alpha power modulation with task showed a strong correlation with behavior for the control group but not for the ADHD group; see Figure 2B. These studies suggest that inattentive ADHD patients do not only suffer from reduced but also from inefficient integration of posterior alpha power as it does not seem to be beneficial for behavior, whereas it is for healthy people.

Studies on the combined ADHD subgroup and neuronal oscillations in task settings are sparse. Recently Karch et al. (2012) found that young adults suffering from ADHD showed increased frontocentral gamma band activity shortly after auditory stimulation followed by a voluntary motor response. Yordanova et al. (2001) found that ADHD children between 9 and 12 years of age showed increased induced fronto-central gamma band responses for right auditory stimulation compared to normally developing children, but not for left auditory stimulation. Lenz et al. (2008) found that children of the combined ADHD group showed enhanced visual stimulus-induced gamma power. While gamma power correlated with long-term memory performance for typically developing children, it did not for the ADHD children. In addition, Mazaheri et al. (2013) tested children of the inattentive subgroup and the combined subgroup and compared these two groups with typically developing children. They found significantly more alpha power to response cues for the inattentive subtypes compared to typically developing children (discussed above), but there was no such effect for the combined group. The combined group, however, showed no significant difference in alpha power from the inattentive subgroup. Also, the combined subgroup showed no correlation between behavior and anticipatory alpha, whereas the typically developing children did. In an earlier study, Mazaheri et al. (2010) investigated the response preparation ability in a perceptual switching task in typically developing children compared to children classified in the combined ADHD group in age range from 8 to 12 years. Typically developing children showed a strong alpha power increase in parieto-occipital cortex in preparation for auditory versus visual stimulation, in line with the idea of shutting down the visual stream when preparing for auditory input. ADHD children, however, showed no such difference, but a frontal theta increase instead. Additionally, although in typically developing children parieto-occipital alpha power was inversely correlated with the behavioral cueing effect in the visual condition, there was no such correlation for the ADHD children. Recently, Lenartowicz et al. (2014) investigated the neuronal patterns in a group of children between 7 and 14 years of age comprised of both the inattentive and combined subgroups. In a working memory paradigm, they found reduced vigilance attributed to a less pronounced alpha depression during encoding (i.e., higher occipital alpha activity) in ADHD children than in typically developing children, but in return a stronger alpha power synchronization during stimulus maintenance in ADHD children. Frontal theta during the maintenance period was also elevated in ADHD children, which they interpreted as a compensation for the lack of vigilance during encoding.

The above studies indicate a versatile interplay of hyper- and hypo-activity in specific phases during a task. In line with Lenartowicz et al. (2014) we propose that this might be anchored to improper preparation for the task, indicated by a lack of modulating anticipatory alpha activity to stimulation. Higher gamma during stimulation and higher alpha and theta during stimulus maintenance might thus be the product of compensation for this improper preparation.

### Cognition in healthy, young adults

Within young healthy adults there is a large spread of inter- and intra-individual differences in both neuronal oscillations and behavior. For example, healthy individuals differ in visual working memory capacity (Luck and Vogel, 2013). Individuals with better working memory capacity have greater frontal theta power during stimulus encoding (Gevins and Smith, 2000). Similarly, individuals with greater pre-stimulus frontal theta power better remembered source context (Addante et al., 2011). Also within subjects, high frontal pre-stimulus theta power was predictive of whether an item was remembered (Addante et al., 2011), as was increased gamma power during stimulus encoding; see Figure 1C (Osipova et al., 2006).

The ability to sustain attention varies among individuals. Individual differences in the degree of alpha lateralization have been linked to differential abilities to ignore the task-irrelevant hemifield (Fu et al., 2001; Haegens et al., 2011a; Horschig et al., 2014) as well as to working memory performance (Sauseng et al., 2009b). Gamma power has been linked to improved attention as well. Apart from the fact that gamma power is commonly increased for attended versus unattended stimuli (Tallon-Baudry et al., 2005; Bauer et al., 2006), individuals with stronger gamma activity have been associated with improved perceptual processing (Jokeit and Makeig, 1994; Fründ et al., 2007). Moreover, the ratio between theta and gamma peak frequency has been successfully linked to short-term memory capacity (Jensen and Lisman, 1996, 1998), which both consistently vary from individual to individual (Kamiński et al., 2011). In future studies, explicit perturbation of the specific neuronal oscillations is required to identify whether they are causally involved in cognition and, if so, the findings can serve to form strong hypotheses on how to augment human cognition.

## Techniques to manipulate neuronal oscillations

In this section we will discuss how to apply our framework in practice. We will combine the fundamental insights of neuronal oscillations from the second section with the studies on different population groups discussed in the third section to answer which oscillatory components might be suitable for optimization and whether and how this optimization could increase cognitive performance. Since there are oscillations-behavior correlations in healthy adults and oscillations-pathology correlations, optimization of neuronal oscillations could lead to optimized behavior. We will introduce different, non-invasive approaches to manipulate neuronal oscillations, outline how these techniques work, and present studies on what has already been achieved. In addition we will suggest steps to fill the gap in current literature on successful augmentation of human cognition.

### Transcranial stimulation of the human brain

**Figure 3:**
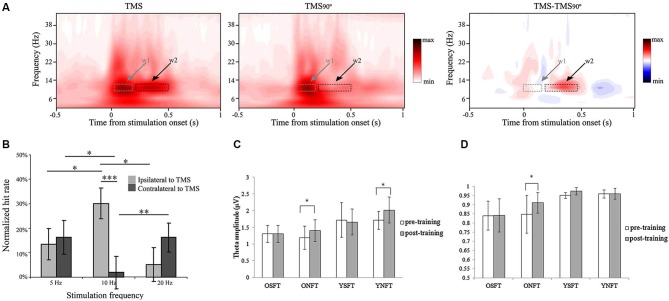
**Examples of manipulating neuronal oscillations and their impact on cognition. (A)** rTMS stimulation at 10 Hz to right parietal cortex results in alpha oscillations outlasting the stimulation period (*t* = 0 s), compared to the control condition of rotating the TMS coil by 90° (TMS90). The condition contrast with other control conditions confirmed the exclusive effect of rTMS at 10 Hz. w1 = time window of the first two pulses and w2 = time window of the last three pulses. Reproduced with permission from Thut et al. (2011b). **(B)** 10 Hz rTMS stimulation, but not 5 or 20 Hz, of parietal cortex ipsilateral to stimulation results in behavioral improvement, whereas contralateral stimulation results in decreased performance (* *p* < 0.05, ** *p* < 0.01, *** *p* < 0.001). Reproduced with permission from Romei et al. (2010). **(C)** Frontal theta neurofeedback training results in increased frontal theta in both young and old adults. OSFT = old subjects, sham feedback; ONFT = old subjects, neurofeedback group; YSFT = young subjects, sham feedback; YNFT = young subjects, neurofeedback (* *p* < 0.01). Taken with permission from Wang and Hsieh (2013). **(D)** Only old adults receiving neurofeedback on frontal theta increased working memory accuracy in a Sternberg task (depicted on the y-axis). Young adults were already performing at ceiling level. (for acronym, see **panel C**; * *p* < 0.01). Taken with permission from Wang and Hsieh (2013).

The most direct way to manipulate neuronal firing is by electrical stimulation to manipulate the neuron’s membrane potential causing it to de- or hyperpolarize. The two common non-physically-invasive techniques to do so are transcranial magnetic stimulation (TMS) and transcranial current stimulation (tCS). TMS utilizes the fact that a changing current in a wire induces a changing external magnetic field that, if in the presence of a conducting material such as neural tissue, induces a secondary electric current in the opposite direction (Pascual-Leone et al., 1999, 2000). This secondary current then affects local membrane potentials. Although the exact mechanism is still not completely understood, it is assumed that TMS pulses primarily influence the axons of both excitatory and inhibitory neurons and might actively elicit action potentials (Dayan et al., 2013). Two basic TMS approaches are commonly used. The “online” approach applies single, double, or brief bursts of pulses, each lasting a few 100 ms, during the task. The “offline” approach applies repetitive stimulation (rTMS) before a task or other measurement (Huang et al., 2005). Depending on the exact pattern and frequency of rTMS, cortical excitability can be facilitated or inhibited for a period outlasting the stimulation itself from 15 up to 90 min (Thut and Pascual-Leone, 2010). However, it has also been suggested that the frequency of stimulation entrains the neuronal oscillations at the stimulation frequency, which outlasts stimulation for a short period of time (hundreds of milliseconds; Thut and Miniussi, 2009). There is some first direct evidence for rhythmically entraining alpha oscillations in the visual cortex by means of brief bursts of rTMS around 10 Hz (Thut et al., 2011b and Figure 3A), yet stimulation not necessarily in the alpha frequency range can induce an alpha power increase (Thut and Pascual-Leone, 2010). Several studies have provided convincing indirect evidence for rTMS entrainment by its impact on behavior (Romei et al., 2010 and Figure 3B; Klimesch et al., 2003; Sauseng et al., 2009a).

The basic principle of tCS is that a weak electrical current is established between an anode and cathode, thereby altering neural membrane potentials. Importantly, because of the weak electrical current, tCS cannot trigger action potentials but rather slightly facilitates or inhibits spontaneous neuronal firing, depending on its polarity (Dayan et al., 2013; Reato et al., 2013). Transcranial current stimulation can be applied in two different ways: transcranial alternating current stimulation (tACS) and transcranial direct current stimulation (tDCS). While with tDCS the polarity between anode and cathode stays constant (see Priori, 2003, for a review), with tACS the polarity constantly changes, thereby producing an alternating flow of current similar to an oscillation (Antal and Paulus, 2013; Herrmann et al., 2013; Reato et al., 2013). Sometimes tACS is used in conjunction with a DC offset. In that case, the polarity between anode and cathode stays fixed, as the current strength oscillates around the DC offset instead of zero. Consequently current only flows in one direction, from the cathode to the anode, which is in contrast to the classical tACS definition, where not only current strength but also direction changes throughout each oscillatory cycle. Here, we will explicitly clarify if we refer to tACS studies with a DC offset.

In the past a number of studies has shown improved cognition or performance in tasks using tCS or TMS (Meinzer et al., 2012, 2013; Snowball et al., 2013; Vollmann et al., 2013; Coffman et al., 2014; Luber and Lisanby, 2014; Schutter, 2014). However, most of these studies did not use tCS or tACS to stimulate neuronal oscillations or concurrently recorded them by EEG or MEG. In the following we will summarize a number of studies that used tACS or TMS to stimulate in the alpha, theta, or gamma range.

Although this is a relatively recent field, a number of studies has demonstrated that stimulating in the alpha range results in sustained alpha power increases in the cortex. Zaehle et al. (2010) showed that applying tACS offline for 10 min at the subject’s individual peak alpha frequency over left and right posterior cortex resulted in an increase in alpha power for at least 3 min after the stimulation. Neuling et al. (2012) used tACS with a DC offset to entrain a 10 Hz rhythm during the task. After 3 blocks of 7 min stimulation, alpha power increased post-stimulation during a measurement lasting 3 min. In a more recent study, Neuling et al. (2013) found that the increase in alpha power using tACS without a DC offset lasts for up to 30 min. However, neither of these two studies found an influence of the power increase on behavior.

In contrast, several studies have found effects of entrainment in the alpha band on behavior using TMS, albeit they do not *per se* show that they were successful in changing cortical alpha power. Hamidi et al. (2009) applied rTMS at 10 Hz in parieto-central regions during the 3 s maintenance period in a delayed-match-to-sample task and found beneficial effects for a spatial working memory task, but not for a non-spatial working memory task. This is in line with the idea that high alpha power is needed during working memory maintenance to block additional incoming information and that the dorsal (parietal) regions encode spatial information but inferior (temporal) regions encode identity information (Goodale and Milner, 1992). Interestingly however, while Hamidi et al. (2009) did not find a change in alpha power due to TMS at the group level, they did find an across-subjects correlation of alpha power with behavior. Romei et al. (2010) found that entraining the alpha rhythm ipsilateral to the attended hemifield increased perceptual accuracy, whereas entraining alpha contralaterally decreased accuracy, strongly in line with the idea that strong alpha inhibits processing (Figure 3B). However, the effect of TMS stimulations on neuronal oscillations was not concurrently assessed by EEG, so it remains to be investigated whether the stimulation also resulted in changes in cortical oscillations.

Brignani et al. (2013) also tried to increase the ipsilateral alpha rhythm but with tACS to parieto-occipital cortex. Surprisingly, they found no spatially specific effect but a general task impairment during a covert spatial attention task. This might have been caused because the stimulated area using tCS is widespread and not easily inferable from the placement of the anode and cathode (Manoli et al., 2012; Bai et al., 2014). Also in this study, no concurrent EEG recordings were done, so the effect of stimulation on cortical oscillations could not be directly assessed. These studies show that different brain regions are affected differently by different techniques on different target areas and that precise *a priori* hypotheses and knowledge about the brain region to be stimulated are necessary for successful augmentation of human cognition. *A priori* computational modeling of the stimulation protocol using advanced physical models has recently been demonstrated (tCS: Manoli et al., 2012; Bai et al., 2014; TMS: Bijsterbosch et al., 2012; Janssen et al., 2013; Wagner et al., 2014). This could help in applying protocols and stimulation electrodes and thereby aid in studying whether modulation of cortical alpha power in specific regions has behavioral consequences.

Fewer studies are available which attempt to induce theta oscillations. Meiron and Lavidor (2014) applied tACS in the theta range to bilateral frontal cortex during a working memory task and found an improvement in online working memory capacity. Jaušovec and Jaušovec (2014) used tACS in the theta range but around left parietal cortex and showed that this leads to an improved memory working memory span. Jaušovec et al. (2014) replicated their finding on stimulating the theta rhythm, but this time in bilateral parietal cortices and replicated the effect on improved working memory capacity. They also report a null-effect for stimulating frontal cortex. While these findings suggest that entrainment of the theta rhythm is spatially specific and results in increasing working memory, they did not report oscillatory responses to assess whether the theta power was successfully increased over the stimulated area. In addition, none of these studies used control frequencies to show a frequency specific effect. Further studies are required to measure spatial and spectral specificity, disentangle which part of working memory is affected, and show convincingly that theta oscillations are entrained or at least modulated during stimulation.

In the gamma range, Chanes et al. (2013) showed a differential effect on performance in a perceptual detection task for 30 Hz stimulation versus 50 Hz stimulation using TMS to right frontal eye fields, where the former enhanced perceptual sensitivity and the latter shifted the response criterion. Using tACS, Santarnecchi et al. (2013) showed that entraining gamma around 40 Hz at the left mid-frontal gyrus improved performance in conditioning and reasoning tasks. Laczó et al. (2012) showed that gamma stimulation to early visual cortex around 60 Hz but not at 40 or 80 Hz resulted in a lower contrast detection threshold. Importantly, none of the stimulation frequencies resulted in behavioral changes in spatial detection tasks. While showing promising results, studies on rhythmically entraining the gamma range are sparse and differ in exact frequency range and spatial location. Reproductions of these findings are required in order to draw definite conclusions on where and how to stimulate to improve what aspect of perceptual performance by gamma entrainment.

All of the above reviewed literature was on healthy, young adults and not on other target groups. Given the partly incomplete literature and understanding of TMS and tACS, it remains to be tested whether such hypothesis-driven brain stimulation shows success in the elderly and ADHD patients, and whether this results in a reduction of cognitive problems.

### Hypothesis-driven brain computer interfacing and neurofeedback

Brain-computer interfacing (BCI) commonly refers to the technique to use a signal measured from the brain to control a computer or a machine without the use of the peripheral muscle system (Wolpaw et al., 2002). This requires the user’s awareness of the ongoing brain activity, which is made perceivable by means of visual, tactile, or auditory feedback (van Gerven et al., 2009a). The original goal of BCI was to provide the user with an additional output channel for the purpose of communication.

Neurofeedback serves a different purpose by similar means. The goal of neurofeedback is to make the users aware of their brain activity to learn to enhance or decrease the fed back aspects, e.g., theta power, and thereby alleviate pathological symptoms, such as in ADHD (Lubar et al., 1995; Fuchs et al., 2003). An unfortunate major criticism in the field of neurofeedback, however, is often the absence of control conditions, significant effects, a scientifically grounded hypothesis, or reproducibility (see Vollebregt et al., 2014). Recently it has been proposed to start using hypothesis-driven BCI to improve subsequent behavior (Jensen et al., 2011), on which we will now elaborate.

Hypothesis-driven BCI (hdBCI) refers to the idea that insights from fundamental and cognitive neuroscience are applied to infer a robust and reliable control signal to improve cognition. Such a control signal must show strong single trial correlation with behavior and must be trainable. As described above, neuronal oscillations have been well studied and show strong correlation with cognition in a multitude of task settings ranging from attention or working memory studies to studies on long-term memory encoding and retrieval. This robustness and reliability allows making a concrete hypothesis to be tested when applying neurofeedback techniques. We suggest using a control signal that shows strong correlations with behavior, for example frontal theta during working memory or alpha lateralization during covert visual spatial attention tasks, and train the subjects to gain awareness and control of that signal. Before and after hdBCI training, a number of behavioral tasks orthogonal to the hdBCI task should be used to assess whether the neurofeedback training translates to subsequent improvements in cognition. The neuronal oscillation and the hdBCI paradigm need to reflect the underlying cognitive mechanism to be trained. For example, we described problems with general attention and reduced ability to suppress distracting stimuli in the elderly or in ADHD patients - a functional role that alpha oscillations are supposed to fulfill. The aim of hdBCI training is to study whether the training effect on oscillatory power brings along changes in behavior, in particular ideally, that has been shown to be correlated to that oscillation. If subsequent behavioral improvements in untrained, but related tasks are found, this would serve as a strong case that neuronal oscillations are causally involved in cognition. In the following we will review studies that can serve as templates for how hdBCI training can be used to study the functional role of neuronal oscillations.

While alpha-based neurofeedback has been shown to modulate not only the alpha power but also performance on a mental rotation task (Hanslmayr et al., 2005b; Zoefel et al., 2011), we link the findings of alpha power’s role in inhibition of distracters (see Section Functional Role of Neuronal Oscillations) to hdBCI training with the goal of improving resilience to distraction. No such study has been conducted so far. If successful, one might ask whether the same rationale can be used for treating inattentive symptoms of the elderly or ADHD patients. As a concrete example, one could study whether the alpha lateralization pattern during covert spatial attention can be trained and strengthened. Alpha lateralization has been studied intensively in the past and has been shown to be a reliable control signal for BCI (Kelly et al., 2005; van Gerven and Jensen, 2009; van Gerven et al., 2009b; Bahramisharif et al., 2010; Tonin et al., 2012). In healthy young adults, high memory load coincided with strong alpha lateralization during a covert attention working memory task—an aspect that was missing in the elderly (Sander et al., 2012b). An hdBCI can consist of training alpha lateralization in the elderly asking whether strong alpha lateralization will be of beneficial nature as observed in young adults and whether a correlation between alpha lateralization and high memory load will reemerge. In a similar line a lack of maintaining alpha lateralization has been found in inattentive ADHD patients (ter Huurne et al., 2013). hdBCI training on maintaining a high degree of alpha lateralization could help to restore this ability. Again, a correlation between behavioral performance and degree of alpha lateralization was observed in healthy adults, but lacking in ADHD patients. The most critical question is, if the ability to maintain a high degree of alpha lateralization is restored in inattentive ADHD patients, will the underlying mechanisms leading to the alpha power shifts be beneficial for their performance again? No studies in this direction have been pursued so far, but they would elucidate the functional role of alpha oscillations.

Complementarily, during working memory paradigms it has been shown that frontal theta reflects the memory load in healthy individuals (Jensen and Tesche, 2002), but no such relation was found in the elderly (McEvoy et al., 2001). One might ask whether training the elderly to increase their frontal theta proportional to memory load during a working memory task results in improved working memory performance. Recently, Enriquez-Geppert et al. (2014) and Wang and Hsieh (2013) provide preliminary evidence that frontal theta neurofeedback training does work. The latter shows that both young and old participants learned to increase frontal theta by neurofeedback in contrast to control groups (see Figures 3C,D). In addition elderly receiving frontal theta neurofeedback training showed improved performance in a subsequent working memory task in contrast to the control group. However, they do not show whether the neurofeedback training restored the correlation between memory load and frontal theta in the elderly. Also their study was confounded by several other issues, for example young, healthy, adults were performing at ceiling level already before the training, which resulted in a null-effect training for them. Thus, this study is inconclusive on whether frontal theta training increases working memory capacity in young, healthy adults. For ADHD patients it has been found that frontal theta is already relatively high compared to young healthy adults, e.g., during working memory encoding, which might be caused by a lack of proper preparation to the task, quantified by weaker anticipatory alpha oscillations in posterior regions (Lenartowicz et al., 2014; see also Section Population Target Groups and Their Neuronal Signatures). Given all reviewed literature, it seems plausible that an additional increase in frontal theta during encoding or increase in posterior alpha in anticipation of the stimulus would further boost working memory performance.

Neurofeedback on gamma oscillations has been studied by Keizer et al. (2010), who found that successful increases in gamma band power in young, healthy adults correlated with an increase in fluid intelligence and reduced cost of feature binding reflected in the lower reaction times. In line with the current view on feature-binding problems in the elderly (cf. Sander et al., 2012a), Staufenbiel et al. (2014) used neurofeedback training of gamma oscillations in the elderly. Although the neurofeedback training resulted in increased gamma, they failed to show a beneficial nature of this training for fluid intelligence, working memory, and quality of life. However, these studies did not feedback gamma power during stimulation, but during resting state. It might have been beneficial to give feedback of gamma power during stimulation, as the neural sources processing stimuli are likely to differ from resting state sources, when no stimulus is being processed. No studies on training gamma band in ADHD patients have been conducted.

For a more complete overview on neurofeedback studies, the interested reader is referred to Gruzelier (2013). In general, we advise that neurofeedback studies should follow hypotheses and paradigms that are more focused and grounded in insights gained from fundamental and cognitive research conducted in the last decades, in particular using information on the functional role of neuronal oscillations.

### Brain state dependent tasks (BSDT): adapting the environment to the user’s mental state

The former approach aimed at shaping the user’s brain activity for optimal stimulus processing or task performance. Recent insight in the field of cognitive neuroscience (see Section Functional Role of Neuronal Oscillations) suggests that we can predict cognitive behavior by neuronal oscillations. This knowledge can be used to adapt the task environment based on the user’s current brain activity to allow for optimal performance. Ultimately this could aid the user to develop an optimal brain state more quickly or efficiently. Specifically, the timing and properties of the task would be determined by an online read-out of the current brain state, as quantified by ongoing neuronal oscillatory activity (Hartmann et al., 2011; Jensen et al., 2011). It is even possible to combine this with active brain stimulation (Silvanto and Pascual-Leone, 2008), which was recently demonstrated by Gharabaghi et al. (2014), who applied TMS and provided haptic feedback according to relevant neural oscillatory activity.

Brain state dependent tasks (BSDT) serve two purposes. First, by adapting the environment to the ongoing brain activity, individual cognition could be improved as described below. Second, BSDT informs the user about his ongoing brain state and rewards the user for a “good” brain state in a similar manner as in BCI. BSDT therefore could help the subject’s ability to modulate his brain activity to reach a certain mind setting, or brain state. BSDT can be used in two complementary manners. First, stimulus presentation can be triggered to ongoing oscillatory activity. For example, it has been found that strong prestimulus alpha power in task-relevant regions negatively affects subsequent stimulus processing (e.g., Ergenoglu et al., 2004; Hanslmayr et al., 2005a, 2007; van Dijk et al., 2008; Mazaheri et al., 2009). In a BSDT paradigm, stimulus presentation could thus be triggered only when alpha power is relatively low, thereby increasing efficiency of stimulus processing. A reverse rationale applies, where the task is to inhibit some aspect of the environment: stimulate during high alpha power in task-irrelevant brain regions. For example, background speech might be distracting when visually learning vocabularies. Thus, in a BSDT environment one could first reward high temporal alpha by removing some artificially-added, auditory distraction while visually presenting vocabularies and, in a second step, additionally only present vocabularies when posterior alpha power is low. This could lead to increased processing of the visually presented vocabularies and also increased inhibition of the distracting auditory noise. To generalize this idea, training could be provided in a variety of tasks where the direction and location of alpha modulation varies; this way, it is the control of alpha that is important and learned, not just a focal or unidirectional lesson which might interfere with other tasks. The subject thereby learns the skill to consciously modulate brain oscillations in similar manner to neurofeedback.

A second manner for BSDT is the fact that based on the activity during stimulus processing or maintenance, subsequent behavior can be predicted, as already proposed e.g., by Mazaheri et al. (2009). For example, increased alpha activity during visual processing (Park et al., 2014) and during the retention interval (Meeuwissen et al., 2011) has been shown to strongly correlate with long-term memory encoding performance. Based on these findings, one could predict which items are most likely to be forgotten and present these items again to the subject in order to facilitate long-term memory performance. In addition, measures correlating with memory load, e.g., frontal theta or occipital gamma for visual items, can be read out to predict the current load. This could also be used to prevent memory overload (discussed in Huggins et al., 2014). This second manner represents an online adaptation of the environment and cannot necessarily be utilized offline or result in an offline skill.

For young, healthy adults, strong correlations between oscillatory power and cognition have been found as reviewed in Sections Functional Role of Neuronal Oscillations and Techniques to Manipulate Neuronal Oscillations. However, in Section Population Target Groups and Their Neuronal Signatures we also review evidence that this correlation seems to be absent in the elderly and ADHD patients. In these population groups, compensatory mechanisms might have taken over the function that some oscillations usually represent. While brain stimulation techniques aim to restore the beneficial nature of these oscillations, BSDT aims to predict cognition. It remains to be tested whether BSDT is beneficial for the elderly and ADHD patients when utilizing hypotheses based on another population group. Both manners of applying BSDT—waiting with stimulation until a good distribution of oscillatory power and predicting subsequent behavior by the distribution of oscillatory power—might prove useless, without a correlation between oscillation and behavior. We would therefore advise to identify neural signatures of the compensatory mechanisms, and find appropriate hypotheses for these population groups, or to manipulate the neural oscillations as proposed in the previous two subsections before applying BSDT in a patient group.

## Practical considerations

In the previous section we made some concrete suggestions on how insights from cognitive neuroscience can be applied to augment human behavior. In this section, we will discuss how to define successful interventions, outline practical considerations about our hypothesis that should be regarded when following above suggestions, suggest alternative approaches, and point to possible pitfalls when applying these techniques in the lab, at home, or when valorizing these ideas for commercial use. For ethical considerations, we encourage the reader to read the Nuffield report on neurotechnology (Nuffield Councils on Bioethics, 2013).

### Defining and assessing success

We have collected supporting evidence for hypothesis-driven approaches to augment human behavior. Although literature on this topic is relatively sparse, studies often differ in crucial aspects such as motivation for the study (i.e., the original hypothesis), methodology (e.g., control conditions, number or duration of training sessions, or the number of hours or days after training to test for long-term effects), and conclusions on how to generalize the findings. The most important question to ask when drawing conclusions about these studies is how best to quantify if the applied technique resulted in augmentation of cognition. This question can be disentangled into two parts.

First, we need to quantify that the augmentation effect is caused by the applied technique and not by confounding other reasons. For example, proper control conditions in neurofeedback settings yield results similar to neurofeedback protocols often used for ADHD treatment (van Dongen-Boomsma et al., 2013; Vollebregt et al., 2014). Thus, special care needs to be taken when attributing the beneficial effect to the applied technique, and that the effect is specific to the modulated frequency-band. As another example, it has been shown that auditory perception of the clicking sound of TMS stimulation alone is sufficient to induce an effect in visual cortex (Romei et al., 2012). Other control conditions aside from sham stimulation, such as cognitive behavioral training (Safren et al., 2010; Strenziok et al., 2014) or physical exercise (Halperin and Healey, 2011; Verret et al., 2012), are often more easily applied than brain stimulation techniques. Furthermore, it is very important to be aware of misleading causes of experimental observations, such as temporal ordering of the tasks; a randomized design order is a crucial part of any paradigm.

Second, we need to define what we mean by augmentation of cognition. We first need to define a baseline level of performance before starting the intervention and we need to show a strong, significant increase from this baseline, above the appropriate control discussed above. Additionally, an improvement in one skill might come at a cost in another skill (Brem et al., 2014; Reinhart and Woodman, 2014). Therefore, one core assertion of successful augmentation of human cognition needs to be measured by transfer learning (Klingberg et al., 2002; Dahlin et al., 2008; Klingberg, 2010), i.e., quantifying how the intervention translates to other domains that were not explicitly trained and tempered by any deficits gained in other domains. In other words, one need to clearly establish the scope of the intervention and what does and does not work.

### The danger of undesired side effects

One important aspect to consider when proposing any study with stimulation is to *a priori* think about which brain region to stimulate and for what purpose. In the vast majority of this article, we talked about how to manipulate neuronal oscillations, but for successful augmentation of human cognition one needs to understand that the human brain is organized into different cortical and subcortical structures and each serves multiple, partly overlapping functionalities. When considering augmenting human cognition, one needs to be precise on which part of human cognition. Let us take the example of increased alpha power in task-irrelevant regions. Firstly, one needs to define “task-irrelevant”; for example, in a covert visual spatial attention task, the posterior ipsilateral hemisphere is “task-irrelevant”, but not the contralateral hemisphere (Worden et al., 2000; Thut et al., 2006; Kelly et al., 2009). Secondly, the natural region(s) exerting modulation of the task-irrelevant region(s) should be noted; for example, it has been found that the intraparietal sulcus and the frontal eye fields exert top-down control on posterior areas and that stimulating them has consequences for posterior alpha and subsequent behavior (Capotosto et al., 2009, 2012; Sauseng et al., 2011). Thirdly, stimulation of the “wrong” brain region can lead to unexpected, reversed effects, i.e., where stimulation of one brain region is beneficial for one task and impedimental for another task (Romei et al., 2010; Iuculano and Cohen Kadosh, 2013); for example, when conducting a purely auditory task, the whole visual cortex becomes “task-irrelevant”. Note that the anatomical precision of non-invasive EEG recordings is not high enough to verify the spatial specificity of the measured oscillations. While source reconstruction techniques can increase spatial certainty beyond sensor level information, invasive recordings are necessary for precise spatial localization.

Additional side effects can arise from improper task settings. For example, one might think to save time by concurrently testing for items in memory while memorizing new items. This, however, has been found to be inefficient and led to deteriorated memory performance (Huijbers et al., 2009). In addition, the functional hypothesis has to be correct and grounded in previous findings. For example, while many studies have convincingly related alpha power with inhibition of task-irrelevant regions, this might not hold true for all brain regions (Mo et al., 2011). Thus the exact experimental paradigm has to be vigorously thought through, which requires intensive knowledge from an expert in the field of cognitive neuroscience and/or brain stimulation or hdBCI techniques. Therefore, one needs to be sure which region requires which treatment (e.g., excitation is distinct from release from inhibition) in order to augment cognition successfully without burdensome side effects.

Non-optimal stimulation protocols and task settings might not be the only cause for side effects. As the mechanism behind electromagnetic brain stimulation is not fully understood, a number of unforeseen side effects can occur. Manufacturers restrict the maximum amplitude in their amplifier to a rather low value to reduce the possible risks (e.g., infrequent reports of inducing seizures, kindling, mood changes and scalp burnings) which can be further minimized by following general guidelines (Wassermann, 1998; Rossi et al., 2009). In addition, effects of long-term electromagnetic brain stimulation are rarely studied and not well understood. As Antal and Paulus (2013) wrote about the motor evoked potential (MEP): “Increasing the duration of tDCS results in a prolongation of the induced aftereffects (Nitsche and Paulus, 2000) up to about 13 min whereas doubling the 13 stimulation to 26 min inverses MEP aftereffects into inhibition (Batsikadze et al., 2013). It is unclear if this can be translated to tACS, too”. While an aftereffect reversal is an obvious crucial side-effect and would be undesired, e.g., in the case of attention boosting, even stronger side effects might occur, especially due to the unknown long-term effects on plasticity and anatomical and functional connectivity. Plastic white matter changes in humans have been found following behavioral training (Zatorre et al., 2012; Sampaio-Baptista et al., 2013), and there is some preliminary evidence for white matter changes following electric stimulation (Allendorfer et al., 2012) and neurofeedback (Ghaziri et al., 2013).

### Individual differences

Cognitive neuroscience aims to infer general mechanisms of the brain by studying a subgroup of some homogenous population. Significant statistical tests using random effects analysis then allow making inference from the subgroup to the population. However, finding a group level effect does not automatically mean that all individuals show the same effect, or even a significant effect. An example is found in the strength and adaptation of alpha lateralization (e.g., Händel et al., 2011; Horschig et al., 2014). Inter-individual variability has extensively been described as an issue in the field of BCI, where it has been found that about 1/5 of all individuals are unable to gain control over the control signal (Dickhaus et al., 2009; Vidaurre and Blankertz, 2010). A similar kind of inter-subject variability is reported in tCS techniques, which might arise due to a multitude of factors (Horvath et al., 2014; Krause and Cohen Kadosh, 2014). Thus, while we are proposing that these techniques can be used to decrease interindividual variability, it might be that interindividual variability requires different solutions for different subjects, e.g., different frequency bands or a different location of stimulation sites.

### Alternative aspects of neuronal oscillations to utilize

In this review, we focused on the region-specific power of neuronal oscillations because, as discussed in Section Functional Role of Neuronal Oscillations, they are a robust read-out of the brain state and show strong across-trials correlations with behavior. However, investigating the power of neuronal oscillations is not the only means of quantifying electrophysiological data. The alternatives we discuss below, as well as others not mentioned, may also provide a handle with which to augment cognition via manipulation as discussed in Section Techniques to Manipulate Neuronal Oscillations, if a suitable hypothesis can be formed linking neural activity to behavior. We believe however that our main point has been sufficiently illustrated by the examples (i.e., oscillatory power and patient groups) used.

One natural alternative to power is the phase. In particular, the phase of the alpha and theta oscillations has been studied intensively in the past (Buzsáki and Draguhn, 2004; Montemurro et al., 2008; VanRullen et al., 2014). Recent evidence suggests that specific phases are more optimal for specific tasks (Gho and Varela, 1988; Kruglikov and Schiff, 2003; Mathewson et al., 2009). For example, ignoring a distracter is more successful in a specific phase of alpha oscillations than in its opposed phase (Bonnefond and Jensen, 2012). Interestingly, it has also been shown that tACS can be used to gain control over the phase of an oscillation (Helfrich et al., 2014; Jaegle and Ro, 2014; Strüber et al., 2014; Zanto et al., 2014), which has corresponding, orthogonal behavioral effects. Recent studies also indicate that a visual stimulus regularly flickering at 10 Hz can entrain alpha oscillations in visual cortex outlasting the stimulation period and also that subsequent behavior was modulated in a phasic manner according to the phase of the stimulation flickering (Mathewson et al., 2012; de Graaf et al., 2013; Spaak et al., 2014). In conclusion, studying the phasic nature of oscillations will provide further insight into human perception and provide important, additional information for brain stimulation techniques, hypothesis-driven brain-computer interfaces, and BSDT.

Furthermore another quantification of oscillations includes cross-frequency phase-amplitude coupling, whereby the phase of a slower oscillation is linked to the amplitude of a faster oscillation. For example it has been shown that gamma power waxes and wanes with the phase of alpha or theta oscillations (Canolty et al., 2006; Jensen et al., 2012; Bonnefond and Jensen, 2013; Lisman and Jensen, 2013; Roux and Uhlhaas, 2014). This also has important implications for behavior as, already discussed briefly in Section Functional Role of Neuronal Oscillations, the ratio between a theta cycle and a gamma cycle has been suggested to determine working memory capacity (Jensen and Lisman, 1996, 1998; Kamiński et al., 2011). This knowledge could be used to optimize cross-frequency coupling in order to study its direct effect on behavior.

In addition, we also chose not to focus on connectivity between brain regions, which can be a phase adjustment across regions (Varela et al., 2001; Gross et al., 2004; Fries, 2005; Sauseng and Klimesch, 2008) or a simultaneous power adjustment (e.g., Mazaheri et al., 2009). For example, several studies found a different functional connectivity pattern in the elderly compared to healthy, young adults (Hogan et al., 2011; Onoda et al., 2012; Geerligs et al., 2013, 2014; Oh and Jagust, 2013; Waring et al., 2013). In children with ADHD compared to typically developing children the pattern of functional connectivity is also different (Murias et al., 2007; Mazaheri et al., 2010). Further evidence for the importance of functional connectivity comes from TMS/tCS studies showing that not only is the activity in the stimulated region modified, but also connectivity of the stimulated region to other regions (Strens et al., 2002; Polanía et al., 2012a; Veniero et al., 2013; Shafi et al., 2014) and in some cases this altered connectivity correlated with task modulations (Vidal-Piñeiro et al., 2014) or improved/altered behavior (Lee and D’Esposito, 2012). As the human brain is a huge network of neurons, it seems logical that an optimization of functional connectivity within and across brain-regions is a crucial aspect to optimize human behavior. However, we have just begun to understand the mechanisms behind inter- and intra-regional coupling (Felleman and Essen, 1991; Varela et al., 2001; Callaway, 2004; Fox et al., 2005; Canolty et al., 2006; Lakatos et al., 2008; Schroeder and Lakatos, 2009; Maier et al., 2010; Haegens et al., 2011b; Spaak et al., 2012), so we still have a long way to go until we fully grasp the effect that artificially manipulating functional connectivity has on the human brain and human cognition.

### Problems of usage at home

Applying some of these techniques at home can be a challenge on its own. The most obvious question to ask is whether the proposed techniques can be used alone at home or whether an expert, e.g., a neuroscientist or a physician with proper training, should be visited. Currently, there is a trend in crowd funding and open source projects, which allow individuals to propose and share ideas. This has led to projects such as OpenrTMS^2^ or OpenBCI,^3^ which in theory allow everyone to create their own TMS or BCI protocol. As the human brain is overly complex, however, special care has to be taken on this path of neuro-hacking.^4^ Obviously side-effects, as discussed above, are likely to occur during improper application. Apart from that, additional problems can be expected when applying the discussed brain augmentation techniques at home, e.g., motivational reasons can cause irregular, inefficient use and eventually lead to stopping the treatment. In addition, environmental noise may influence the measurements at home differently than in a well-controlled laboratory. Studies in the lab under special, controlled circumstances might not easily transfer to use at home or other situations of daily life (see e.g., Vaughan et al., 2006). Therefore and especially due to medical reasons we would propose that, first, more research is required to investigate side-effects and long-term effects. Second, application should only be administered by trained experts. Only in the far future does usage at home seem realistic.

### Considerations for successful valorization and commercialization

The above suggestions and hypotheses might seem like a great promise for augmentation of human behavior, so it is natural that commercial companies will pick up on these ideas in the near future. However, when considering augmentation of human behavior from a commercial perspective, there are additional considerations apart from those already discussed, foremost safety issues. Modern advertisements focus on short, catchy messages to attract potential customers (Dahlén and Rosengren, 2005; Kohli et al., 2007). The human brain, however, is a complicated machine and we are just beginning to understand its fundamental mechanisms and functions. Companies have to take responsibility for the products they sell and be wary of linking false promises to their commercial products. Also, companies need to be explicit to the customer on the consequences of not using devices properly as intended. Yet, there is no consensus on proper use of brain stimulation technique. Thus, extreme caution needs to be exercised before bringing a product onto the market. We therefore advise any company with serious perspectives on developing and selling these devices to collaborate strongly with established neuroscientists in the field and ethical committees and to conduct extensive studies on their products.

## Conclusions

In this review, we started from the hypothesis that neuronal oscillations serve as strong neuronal correlates of behavior and are involved in human cognition. We reviewed this hypothesis in light of three target groups: the healthy elderly, ADHD patients, and healthy young adults. Most of the evidence supporting our hypothesis stemmed from studies on healthy young adults showing reliable correlations between the power of the oscillations and cognitive aspects of human behavior, e.g., working memory capacity or detection accuracy. Our hypothesis was corroborated by brain stimulation studies using TMS and tACS showing a strong link between the strength of induced neuronal oscillations and behavioral performance (see Section Techniques to Manipulate Neuronal Oscillations).

This hypothesis has important implications for the realm of cognitive neuroscience: if an oscillation is causally involved in cognition, a manipulation of that oscillation has to lead to a subsequent change in behavior. Such a strong, functional hypothesis helps to drive the field of cognitive neuroscience forward in understanding how the human brain functions.

Further, we hypothesized in Section Population Target Groups and Their Neuronal Signatures that the elderly and ADHD patients suffer from a lack of integrating neuronal oscillations properly, due to an absence of correlation with behavior, and that compensatory mechanisms could have taken over the functional shaping of cognition. We further hypothesized that manipulating neuronal oscillations in these target groups might restore their beneficial nature. This is a far-fetched claim without any empirical evidence and should be further studied. First of all, we need to ask whether the absence of the correlation between neuronal oscillations and behavior still allows the control and modulation of oscillations. Further, if these target groups could increase some neuronal oscillation during tasks, would that have behavioral consequences? Although we hypothesized that there would be, even if there would not be a behavioral benefit, we would gain important, fundamental insight in different neuronal mechanisms to shape human cognition.

In sum, we conclude that many more studies need to be conducted and reproduced, in both young, healthy and other target groups, to elucidate the role of and effects of manipulation of neuronal oscillations on behavior. Further insight from fundamental research into neuronal mechanisms is required to develop robust products for augmenting cognition.

## Conflict of interest statement

The authors declare that the research was conducted in the absence of any commercial or financial relationships that could be construed as a potential conflict of interest.

## Footnotes

^1^Note that we focus on power of the oscillation and do not cover its phasic role. Other frequency bands, such as delta (1–3 Hz) or beta (13–30 Hz) oscillations, or slow cortical potentials lie outside the scope of this article. Some alternative approaches are shortly discussed in Section Alternative Aspects of Neuronal Oscillations to Utilize.

^2^http://open-rtms.sourceforge.net/

^3^http://www.openbci.com/

^4^http://en.wikipedia.org/wiki/Neurohacking
